# Prospective affirmative therapeutics of cannabidiol oil mitigates doxorubicin-induced abnormalities in kidney function, inflammation, and renal tissue changes

**DOI:** 10.1007/s00210-023-02836-4

**Published:** 2023-11-16

**Authors:** Nabil A. Soliman, Samih I. El Dahmy, Amr A. Shalaby, Khadija A. Mohammed

**Affiliations:** 1 Zoology Department, Faculty of Science, Zagazig University, Zagazig, Sharkia Egypt; 2 Pharmacognosy Department, Faculty of Pharmacy, Zagazig University, Zagazig, Sharkia Egypt

**Keywords:** Cannabidiol, Nephropathy, Doxorubicin, Inflammation, IL-6, DNA

## Abstract

Nephropathy is the decline in kidney function. A promising treatment for numerous types of illness is using natural materials as natural chemical compounds. The inquiry was conducted to investigate cannabidiol (CBD) potential for renal syndrome protection. The five equal groups of fifty male Sprague-Dawley rats weighing 150 ± 25 g each were designed; group I received distilled water orally, while group II got an intraperitoneal injection of doxorubicin (18 mg/kg bwt). Group III received CBD (26 mg/kg bwt) orally, while group IV received 1 ml of CBD (26 mg/kg bwt) and group V received trimetazidine (10 mg/kg bwt), in addition to a single intraperitoneal dose of doxorubicin (18 mg/kg bwt) on the 11th day for both groups (IV, V). The administration of CBD (26 mg/kg bwt) led to a noticeable improvement in oxidative stress parameters (SOD and GSH) in rats by significantly lowering enzyme activity (ALT and AST), as well as serum creatinine and urea, IL-6, and MDA, confirming the anti-inflammatory accuracy of CBD linked to significant lowering to IL6R DNA frequency concentration in line with histopathology results. As a result of its anti-inflammatory and antioxidant capabilities, cannabidiol may have protective quality, and CBD medication could be related to controlling renal problems.

**Supplementary Information:**

The online version contains supplementary material available at 10.1007/s00210-023-02836-4.

## Introduction

Ailments of the kidneys are significant public health arguments, resulting from external chemicals like natural toxins and pharmaceuticals, which can eventually cause numerous renal syndromes (Mohammed et al. 2021). An unexpected and frequently reversible decline in kidney function, reflected by an increase in creatinine or a decrease in urine volume, is referred to as acute kidney injury or renal failure. Chronic kidney disease (CKD) is caused by an irreversible loss of kidney cells and nephrons, and leads to persistent kidney dysfunction. Kidney failure is caused by nephrotoxicity, which causes the kidneys to be unable to clear blood urea, creatinine, and electrolytes, which build up in the blood (Kellum et al. 2021; Goyal et al. 2023).

The clinical use of some therapeutic or diagnostic agents, such as antineoplastic drugs, antibiotics, immunosuppressive agents, non-steroidal anti-inflammatory drugs (NSAIDs), and contrast agents, is restricted due to drug-induced nephrotoxicity (DIN), which is a major cause of kidney damage and is associated with high mortality and morbidity. However, recent research has revealed that many natural products (NPs), including phytochemicals, various plant extracts, herbal combinations, and NPs derived from animals, converse protective effects against DIN through multiple therapeutic mechanisms, including inhibition of oxidative stress, inflammation, apoptosis, necropsies, regulation of autophagy, and maintenance of polarity (Gao et al. 2021).

Doxorubicin, an anthracycline antibiotic, is a highly potent chemotherapy drug frequently used to treat a number of malignancies. Even though DOX has strong anticancer properties, its clinical application is constrained by unfavorable side effects such as nephrotoxicity, hepatotoxicity, cardiotoxicity, and gonad toxicity, besides damaging effects on a number of tissues (Öztürk et al. 2020; Ali et al. 2021; Alherz et al. 2022, 2023; Gaytan et al. 2023). A well-known rat model of renal failure is DOX-induced nephropathy.

One of the plants grown for its therapeutic benefits for the longest periods is cannabis, or *Cannabis sativa* L. It generates a variety of phytochemicals, including terpenes, flavonoids, and cannabinoids, and is a significant source of cannabidiol (CBD), one of the most promising components isolated from *Cannabis sativa* (Mehrab Valizadehderakhshan et al. 2021; Assadpour et al. 2023). One of the main components of the cannabis plant is cannabidiol (CBD), which is showing great promise as a medicinal agent since it has potent analgesic, anti-inflammatory, anticonvulsant, and anxiolytic properties without having the same euphoric effects as tetrahydrocannabinol (THC) (Boyaji et al. 2020).

Trimetazidine (TMZ) is an anti-angina medicine that promotes more efficient glucose oxidation and adenosine triphosphate (ATP) synthesis as a reference drug by blocking free fatty acid oxidation under ischemia conditions. TMZ may protect these organs from hemorrhagic shock (HS), which compromises kidney and cardiac function (Gabriel et al. 2021). The purpose of the topical trial was to evaluate CBD oil’s capacity to alleviate renal complications convinced by an anthracycline chemotherapy medication (doxorubicin).

## Materials and methods

The inquiry was mainly conducted at the Zagazig University in Scientific and Medical Research Centre (ZSMRC). The International Animals and Use Committee and the ZU-IACUC Committee approved the revised protocol on December 29, 2021, and assigned it the approval number ZU-IACUC/3/F/205/2021.

### Chemical agents

Doxorubicin hydrochloride, 50 mg in a 25-ml vial: 50 mg of doxorubicin hydrochloride in a 25-ml vial Adricin was manufactured by Hikma Specialised Pharmaceuticals, Badr City, Cairo, A.R.E., and was given intraperitoneal in groups II, IV, and V at a single dose of 18 mg/kg bwt (Moustafa and Ali 2021).

Tricardia (20 mg) film-coated tablets of trimetazidine dihydrochloride (TMZ): The TMZ was donated by Rameda, The Tenth of Ramadan for Pharmaceutical Industries and Diagnostic Reagents. The TMZ tablet was crushed in a mortar with 0.06 g acacia gum and then diluted in distilled water to create an oral daily dose of 10 mg/kg bwt for group V (Gabriel et al. 2021).

### Material preparation

Cannabidiol (CBD) was included from Zova.Co., CA, USA, from a San Diego pharmacy. Each 1 ml of the solution contained 10 mg of cannabidiol (CBD), and it was used as a daily dose (26 mg/kg bwt) by adding 20 g of the compound to 100 ml of warm distilled water with 20 g of acacia gum and after mixing to a volume of 1000 ml of water.

### Experimental animals

From the Veterinary Laboratory Animal Farm of Zagazig University, 50 male Sprague-Dawley rats were purchased. Rats were kept in metallic cages at a decreased number (5 rats per cage) and remained in the laboratory temperature and air exposure model. A standard diet (a healthy mouse chow) and unlimited access to water were provided to the calculated rats. Before the ideal conditions began the experimental period, all the animals were maintained under observation and acclimatization for 2 weeks.

### Experimental design

Rats were divided into five equal groups at random, each weighing 150 ± 25 g at the start. For group I (the control group), distilled water was administered orally. Rats in group II were used for doxorubicin collection; they were given distilled water orally for 14 days, and on the 11th day, 16 h later, only a dose (18 mg/kg bwt) of the drug was administered intraperitoneally. Animals in group III were given CBD (26 mg/kg bwt) orally for 2 weeks, and on day 11, a specific intraperitoneal dose of 10 ml/kg bwt of normal saline was provided after a 16-h delay. Group IV was a CBD + doxorubicin group, receiving CBD orally for 14 days and a single intraperitoneal injection of the chemotherapy drug on day 11. Trimetazidine (TMZ) was delivered orally for 14 days (10 mg/kg bwt) before the administration of a single intraperitoneal injection of doxorubicin (18 mg/kg bwt on the 11th day after 16 h), which was given to group V. Rats that had been fasting had their blood collected to prepare serum samples. For histological studies, the kidneys of each group of rats were cleaned in standard cold saline, dried with filter paper, and preserved in 10% formalin-saline.

### Laboratory examination

Blood samples were taken in non-heparinized tubing on the last day of dosing after being lightly sedated with ether. The serum was conserved at − 20 °C after being centrifuged for 20 min at 4000 rpm. Along with the activities of ALT and aspartate aminotransferase (AST), total protein, and albumin, serum creatinine and blood urea were calculated. Tumor necrosis factor-alpha (TNF-α) and interleukin-6 (IL-6) levels, as well as the DNA frequency concentration of the IL-6 gene, are examples of inflammatory markers. Malondialdehyde (MDA), superoxide dismutase (SOD), and reduced glutathione (GSH) of the kidney homogenates were also evaluated in addition to oxidative stress indicators. The whole kidney was removed from each rat in groups after it was sacrificed, cleaned with regular saline, dried with screen paper, and then preserved in 10% formalin-saline at room temperature for histological investigations. Serum creatinine was measured using a fully automated analyzer SAT 450 with Creatinine Jaffe (single reagent) kit with catalog no. R1101022 and blood urea with ultimate single reagent concluded urease-UV fixed-rate (enzymatic method) with catalog no. 320001. The activities of ALT and AST were determined by the kinetic method utilizing once-made packs in conformity with the procedure according to the International Federation of Clinical Chemistry (IFCC) with catalog no. 261002 and 265002 for the ALT kit. Diagnostic determination of albumin was with Spectrum Kit with catalog number R1110021. Tumor necrosis factor alpha (TNF-α) was stately by Elisa kit with cat. no. E0082Hu and interleukin-6 (IL-6) by Bioassay Technology Laboratory ELISA kit with the cat. no. E0090Hu. SOD of kidney homogenate was determined by rat SOD ELISA kit with catalog no. CSB-E08555r; GSH levels were determined using the Cusabio Biotech Company ELISA kit (GSH catalog # CSB-E12144r), and Rat MDA ELISA kit for renal homogenate.

### Quantification of the IL6R gene in DNA frequency concentration

Quantitative polymerase chain reaction (PCR) was performed at the conclusion of the trial (2 weeks after CBD treatment) using the housekeeping gene (β-actin) according to the manufacturer’s instructions. DNA was extracted with whole blood using G-spin™ total DNA Extraction with Lot. No. 105251452 (iNtRON Biotechnology) after being collected with EDTA and stored for extraction at − 80 °C.). For the amplification, use Xpert Fast SYBR (unit) 2X master mix #GE 020.0100. The Master Mix PCR PreMix tubes were filled with template DNA from the five groups as well as the designated primers (reverse and forward), and specific primers (reverse and forward) were added into the Master Mix PCR PreMix tubes. The β-actin forward and reverse primer sequences were TTCCTTCCTGGGCATGGA (length 18) and GAGGAGCAATGATCTTGAT (length 19), respectively. The IL6R (target gene) forward and reverse primer sequences were GTCGCTTTCCCTCTCCG (length 17) and GGAAACCCCAAGGCAAGAGG (length 20) (Nechemia-Arbely et al. 2008; Su et al. 2017). A real-time PCR detecting thermocycler (DTlite 4S1, DNA TECHNOLOGY R&D, and Russia 2014 with ser. no. A7B010) was used to do duplication during amplification.

**Fig. 1:**
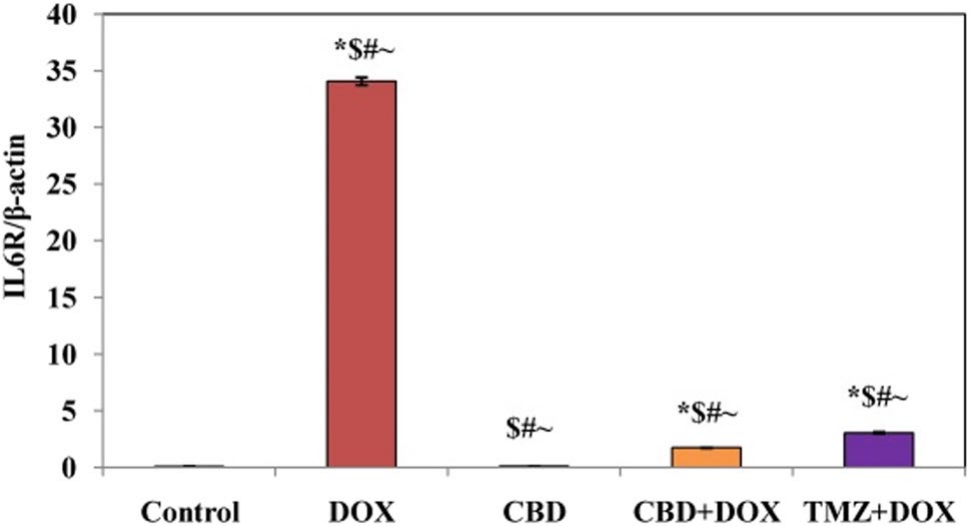
Effects of CBD and drug TMZ on IL6R gene concentration in combination pretreatment, where control, *, significant vs control; $, significant vs DOX, #, significant vs (CBD); and ~ , significant vs (CBD + DOX)

**Fig. 2:**
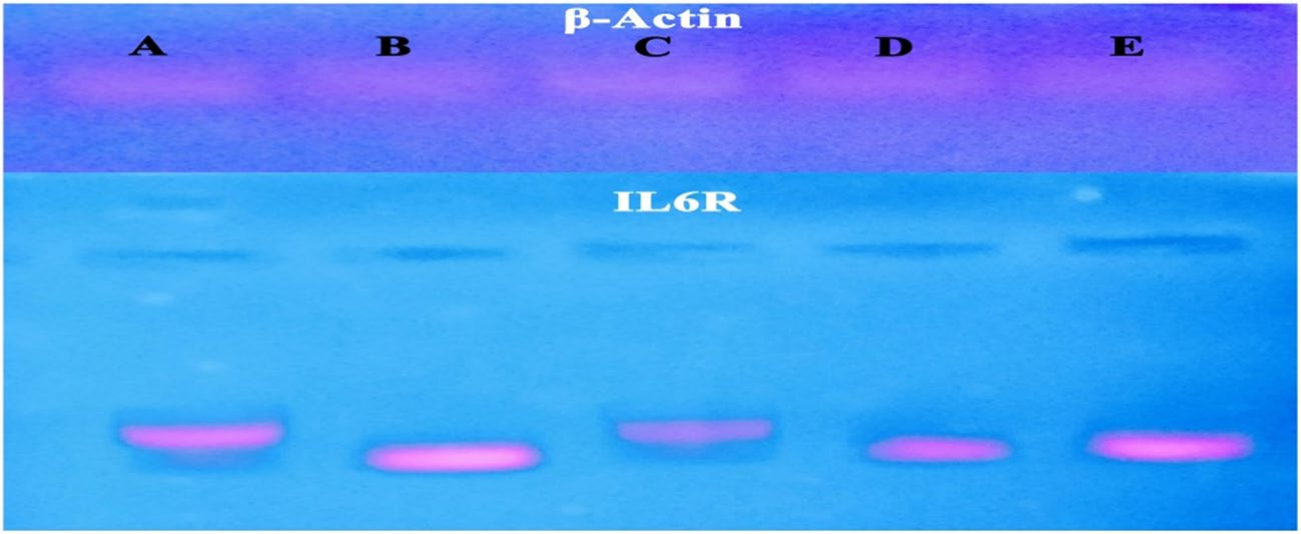
Gel electrophoresis of IL6R gene frequency concentration. Lane A, control; lane B, DOX; lane C, CBD; lane D, CBD + DOX; and lane E, TMZ + DOX

The sample products were performed according to the following amplification program: inactivation of RTase at 94 °C for 2 min; initial denaturation, then 40 cycles of 94 °C for 20 s; 60 °C for 30 s and 72 °C for 30 s; then final extension at 72 °C for 7 min. The products were loaded on 1% agarose gel and were done on device (HOEFE SCIENTIFIC INSTRUMENTS SERIAL NO.95-2032). The target band visualization was with an ultraviolet Trans illuminator (upland, CA91786, USA). Image analysis was by means of Total Lab Quant software to obtain band intensity represented as area underpeak. The final amount of PCR product was expressed as the ratio of the IL6R gene to that of the β-actin gene to account for any differences in beginning amounts of DNA (Figs. 1 and 2). Using the 2 ^-∆∆Ct approach, results were presented as fold-changes from the control group (Livak and Schmittgen 2001).

### Statistical analysis

The mean and SD of the statistical data were used to indicate them using SPSS Statistics 19. ANOVA took the indicators’ levels into account.

## Results

### Cannabidiol (CBD 26 mg/kg bwt) improves renal function and enzyme activity in normal and injured rats

**Fig. 3:**
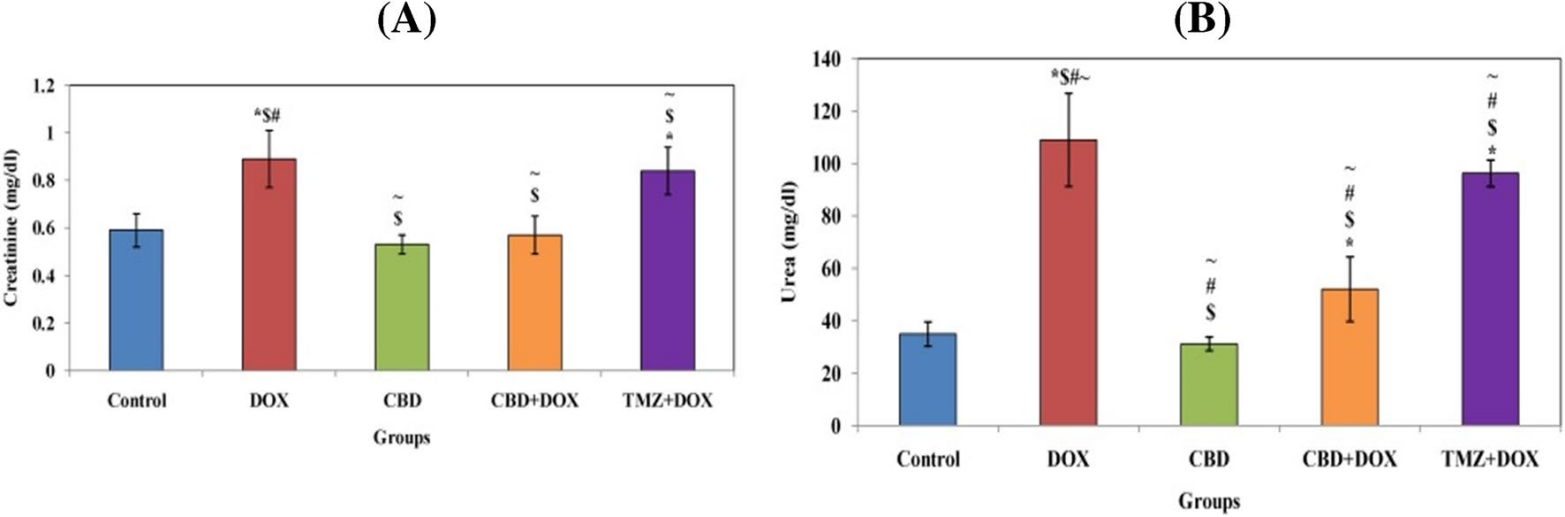
The effects of CBD and drug TMZ on kidney function in combination pretreatment. **A** Creatinine. **B** Urea where control. *, significant vs control; $, significant vs DOX; #, significant vs (CBD); and  ~ , significant vs (CBD + DOX)

**Fig. 4:**
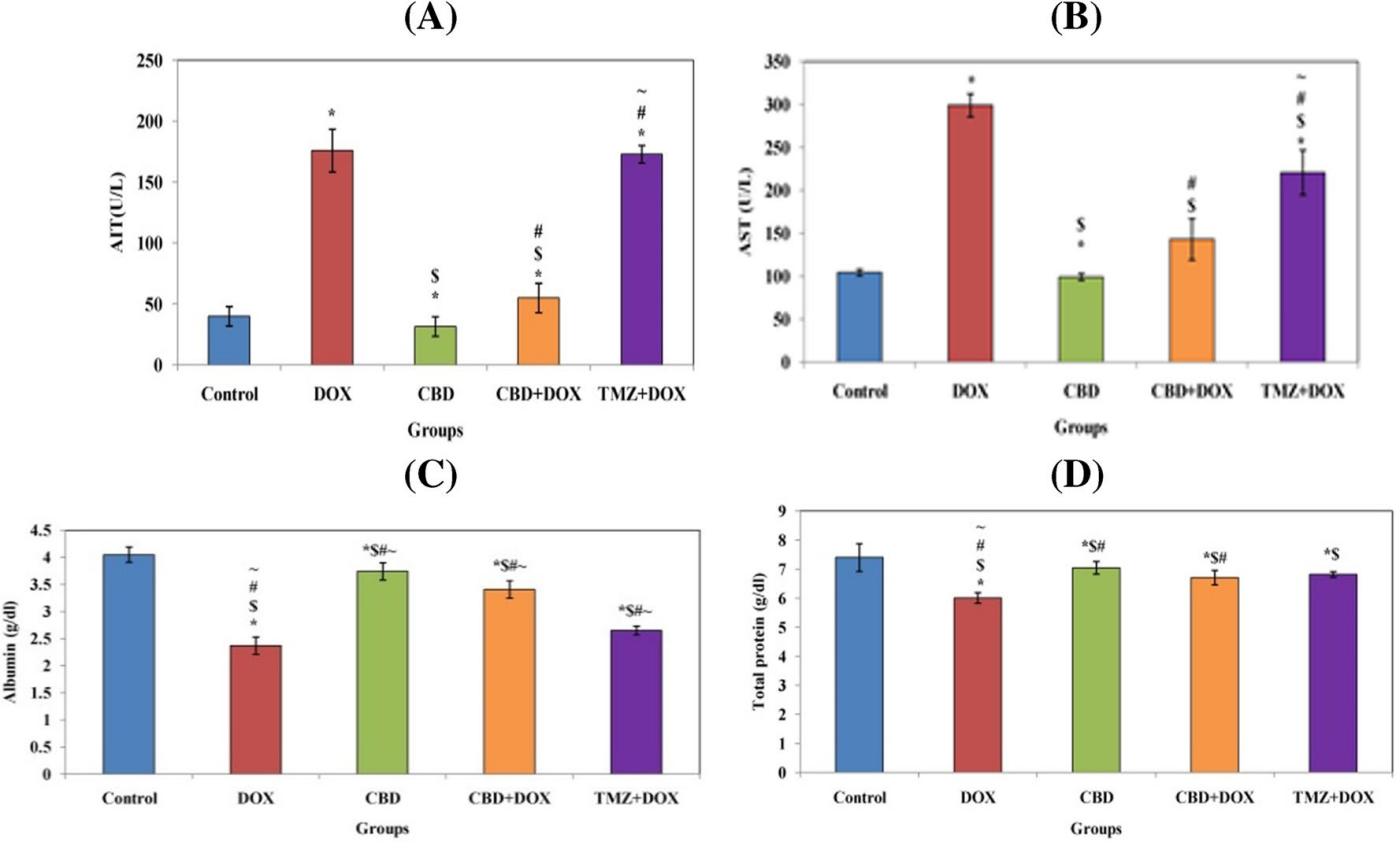
The effects of CBD and drug TMZ on enzyme activity and protein levels in combination pretreatment. **A** ALT. **B** AST. **C** Albumin. **D** Total protein where control. *, significant vs control; $, significant vs DOX; #, significant vs (CBD); and  ~ , significant vs (CBD + DOX)

According to the data shown in Fig. 3, oral administration of CBD as a preemptive measure before receiving a single intraperitoneal dose of doxorubicin has a minor impact on improving kidney function tests, including serum creatinine and urea, a significant value of *P* < 0.0001 in comparison to the DOX group (II). In addition, significant decrease in ALT and AST levels in group IV compared to the DOX group and combination group pretreatment with TMZ (*P* < 0.0001). Then, according to the usual trials, CBD demonstrated decent control. Additionally, albumin and total protein values significantly increased following pretreatment with CBD, whereas they decreased in the DOX group (Fig. 4). According to TMZ, CBD functioned effectively as a reference drug and was comparable to the standard of care.

### The impact of CBD (26 mg/kg bwt) as anti-inflammatory and renal antioxidants

The findings (Table 1) showed a substantial increase in TNF-α levels in the doxorubicin (G2) group (*P* < 0.0001) and an increase in IL-6 (*P* < 0.0001) in comparison to the control group. When compared to pretreatment with the drug TMZ and rats exposed to the toxin in the doxorubicin group, respectively, the time needed for the control group to return to normal values was significantly shortened (*P* < 0.0001). The results (Table 2) show that when the control group received CBD before receiving doxorubicin monotherapy, there was a considerably lower level of MDA and an increase in SOD and GSH in renal homogenates.

**Table 1:** Inflammatory marker values in the control and treatment groups

Treatment	IL-6 (ng/L)	TNF-α (ng/L)
Control	280.46 ± 32.37	29.52 ± 2.11
DOX	393.94 ± 16.51^*^	80.82 ± 1.55^*^
CBD	284.98 ± 21.80^$^	27.85 ± 1.37^$^
CBD + DOX	282.44 ± 18.78^$^	43.18 ± 3.51^*$#^
TMZ + DOX	255.80 ± 15.61^*$#~^	34.99 ± 7.75^*$#~^

^*$#~^Mean ± SD at the same column and bearing different superscripts are significantly different at *P* value, *P* < 0.0001, *P* < 0.01, *P* < 0.05

**Table 2:** Oxidative stress of renal homogenate in the control and treatment groups

Treatment	SOD (U/mg renal tissue)	GSH (ng/mg renal tissue)	MDA (nmol/mg renal tissue)
Control	131.04 ± 4.99	149.43 ± 7.58	2.15 ± 0.91
DOX	39.0 ± 2.79^*$#~^	67.83 ± 6.84^*$#~^	15.08 ± 1.88^*$#~^
CBD	129.70 ± 5.54^$#~^	148.17 ± 3.27^$#~^	2.07 ± 0.75^$~^
CBD + DOX	120.28 ± 1.45^$#~^	121.71 ± 3.58^*$#~^	2.33 ± 0.74^$~^
TMZ + DOX	99.31 ± 6.70^*$#~^	100.08 ± 6.73^*$#~^	9.23 ± 1.09*$^#~^

^*$#~^Mean ± SD at the same column and bearing different superscripts are significantly different at *P* value, *P* < 0.0001, *P* < 0.01, *P* < 0.05

### Histological studies (histopathological studies)

**Fig. 5:**
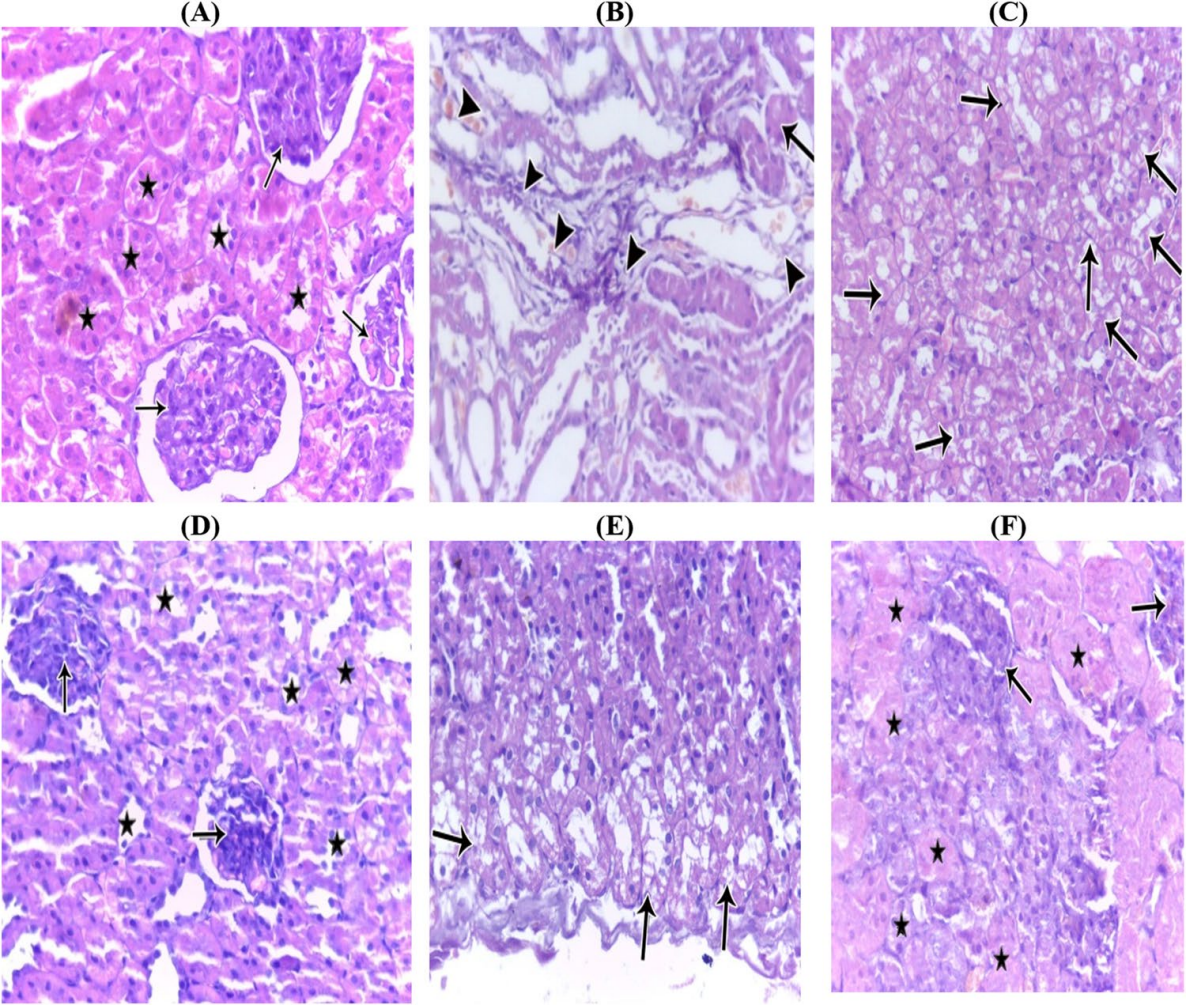
**A** Photomicrograph of control healthy renal tissue of a rat showing normal glomeruli (arrow) and tubules (star) (H&E, × 400). **B** Photomicrograph of renal tissue of a rat under effect of doxorubicin showing trophic glomeruli (arrow) with aggregates of inflammatory cells and areas of hemorrhage (arrowhead) (H&E, × 400). **C** Photomicrograph of renal tissue of a rat under effect of doxorubicin showing severe hydropic degeneration (arrow) of the renal tubules (H&E, × 400). **D** Photomicrograph of renal tissue of a rat under the effect of CBD showing normal renal glomeruli (arrow) and tubules (star) (H&E, × 400). **E** Photomicrograph of renal tissue of a rat under the effect doxorubicin and treated with CBD showing mild hydropic degeneration of the renal tubules (arrow) (H&E, × 400). **F** Photomicrograph of renal tissue of a rat under the effect doxorubicin and treated with TMZ showing marked improvement and nearly normal glomeruli (arrow) and tubules hydropic degeneration of the renal tubules (star) (H&E, × 400)

The CBD and DOX groups (G4) in the current study received CBD treatment first, and then they were given a single intraperitoneal dose of doxorubicin (18 mg/kg bwt). Figure 5E shows mild hydropic degeneration of the renal tubules, in contrast to Fig. 5B, C which shows trophic glomeruli with aggregates of inflammatory cells and areas of hemorrhage and severe hydropic degeneration.

## Discussion

Chemotherapeutic drugs frequently treat many forms of cancer, but they can also have physiological side effects in non-tumor cells and frequently disrupt physiological homeostasis in several organs. This effect on oxidative stress and the generation of free radicals cause side effects of chemotherapy (Ali et al. 2017). Doxorubicin (DOX), an abundant chemotherapy drug, has been connected to several serious adverse effects, including kidney impairment (Su et al. 2015). Nephrotoxicity, also known as renal dysfunction with impaired filtration, reabsorption, and excretion, is one of its most frequent side effects and is linked to a high risk of morbidity and mortality. Nephrotoxicity is a side effect of chemotherapy that affects the kidneys in about 60% of cancer patients (Fukasawa et al. 2014; Ibrahim Fouad and Ahmed 2021).

The current investigation is to evaluate the prospective affirmative therapeutic properties of cannabidiol (CBD) oil and its antioxidant function against renal toxicity in a rat model of doxorubicin (DOX)-induced renal damage using trimetazidine as a reference drug. Blood urea nitrogen (BUN), albumin, and creatinine are reliable indicators of renal dysfunction. However, in the event of renal dysfunction, these processes are disturbed: albumin is excreted in the urine, resulting in reduced serum concentrations, and creatinine and BUN are not filtered correctly, resulting in elevated serum values (Ali et al. 2021).

Our results revealed higher levels of urea and creatinine in the serum of rats given DOX injection which is simultaneous with a reduction in glomerular filtration rate concurrent with tubular blockage and damaged renal tissue in comparison with the control group and combination group with trimetazidine ( El-Sheikh et al. 2012; Liu et al. 2019; Afsar et al. 2020; Asaad et al. 2021) while treatment with cannabidiol affected substantial drop in serum urea and creatinine levels in similarity with Pan et al. (2009) and Hokmabadi et al. (2023). The results are parallel to those of several researchers who discovered that doxorubicin treatment decreased levels of albumin and total proteins while enhancing enzyme activity (ALT and AST) (Mancilla et al. 2019; Moustafa and Ali 2021; Ikewuchi et al. 2021; Saleh et al. 2022). Additionally, the potential impact of CBD treatment caused improvements in ALT and AST (Wang et al. 2017).

According to Chen et al. (2016) and Hegazy et al. (2021), our investigation showed that a single intraperitoneal dosage of doxorubicin that caused kidney damage was the actual cause of the enzyme difference. Apoptosis stimulus, nitric oxide (NO) inflection, and inflammatory stress are the probable causes of the fatal adverse effects of DOX cure (Owumi et al. 2021). The subsequently occurring direct or indirect activation of NO production controls the alleged function of DOX in NOS metabolism. Acute renal failure is a side effect of chemotherapy-induced severe renal tubular abnormalities (Ruggiero et al. 2017). The majority of the mechanisms that control DOX-induced nephrotoxicity have proximal tubule cell-specific targets (Grant et al. 2019; Soltani Hekmat et al. 2021). CBD affects the signaling pathways that alter autophagy, apoptosis, and the suppression of angiogenesis and metastasis in cancer cells (Velasco et al. 2016). Doxorubicin kidney damage is a significant problem in cancer treatment (AlAsmari et al. 2022) and one example of multi-organ damage that is primarily facilitated by the generation of free radicals and ultimately results in membrane lipid peroxidation (Ghibu et al. 2012).

Similar to our investigation results in the DOX group, Afsar et al. (2020) and Mancilla et al. (2019) showed that a reduction in SOD, GSH activity, and MDA level elevation led to the kidney’s ability to scavenge harmful H_2_O_2_ and lipid peroxides. A number of cells, including B lymphocytes, T lymphocytes, myeloid cells, epithelial cells, fibroblasts, endothelial cells, muscle cells, and adipocytes, interact during the complex process of inflammation. These interactions are mediated by membrane-associated molecules, matrix metalloproteases (MMPs), and soluble factors like cytokines, chemokines, and growth factors. Numerous trials have found that Dox increases the production of pro-inflammatory cytokines such interleukin-6 (IL-6), and tumor necrosis factor alpha (TNF-α), as well as causes inflammation. IL-6 is a key performer in the development of cancer, cytokine storms, autoimmune illnesses, and chronic inflammatory diseases (Johnson et al. 2018).

A pleiotropic cytokine called interleukin-6 (IL-6) modulates immunological and inflammatory responses as well as hematopoiesis, metabolism, and organ development. The signalling cascades known as classic and trans-signaling are likely used to distinguish between the several physiopathological processes that IL-6 might simultaneously provoke. Dysregulated IL-6 has been shown to be the root cause of a number of autoimmune and inflammatory disorders, metabolic abnormalities, and malignancies in addition to having other significant physiological roles (Su et al. 2017).

Increased ROS generation after Dox-induced kidney injury has been shown in numerous studies to be essential for initiating the intrinsic apoptotic pathway through mitochondrial instability. This pathway is controlled by the mitochondrial-associated proteins Bax and Bcl-2; a balanced ratio of Bcl-2 to Bax inhibits apoptosis, whereas an imbalance causes cytochrome c leakage into the cytosol and increased membrane permeability, which activates caspase-9 (Casp-9) and caspase-3 (Casp-3). Such activation frequently leads to DNA fragmentation and cell death. The altered levels of renal function indicated that a single doxorubicin injection caused kidney injury (Wu et al. 2021).

Inflammation, hematopoiesis, bone metabolism, and embryonic development are all impacted by IL-6, as are immunological reactions. Chronic inflammation, associated with autoimmune disorders, cancer, and other chronic inflammatory diseases, is caused by IL-6. Chronic inflammation and cytokine storm are uncontrolled inflammatory reactions, in contrast to acute inflammation caused during an immune response and wound healing. The transcription factors nuclear factor-kappa B (NF-κB) and signal transducer and activator of transcription 3 (STAT3), as well as immune and non-immune cells, cytokines like IL-1, IL-6, and tumor necrosis factor alpha (TNF-α), play crucial roles in inflammation. The hyperactivation of NF-κB and subsequent production of several inflammatory cytokines result from synergistic interactions between NF-κB and STAT3. The simultaneous activation of STAT3 and NF-B is because IL-6 is an NF-B target (Shimizu et al. 2021).

For upregulation to the expression of IL6R in a group of doxorubicin in comparison with control and other groups, Nechemia-Arbely et al. (2008) showed that renal autoimmune and inflammatory disorders are associated with local activation of the IL-6 classic and trans-signaling pathway. Under some conditions, kidney resident cells such as podocytes, endothelial cells, mesangial cells, and tubular epithelial cells (TECs) can release IL-6. The only resident cell that expresses IL-6R is the podocyte; all other cells lack this receptor and lack typical IL-6 signaling. Renal IL-6 mRNA expression rose in mice with either AKI or CKD, indicating the kidney is the source of the elevated serum IL-6 levels in the uremic state by our results of upregulation to IL6R frequency concentration. Circulating sIL-6R levels increased in both conditions of CKD and AKI mice (Durlacher-Betzer et al. 2018). In the damage progression, renal IL-6 expression and STAT3 activation considerably increased in renal tubular epithelial cells, indicating active IL-6 signaling. IL-6 can stimulate target cells when combined with a soluble form of the IL-6R (sIL-6R), a method known as trans-signaling, even if the absence of renal IL-6 receptors (IL-6R) prevents the activation of conventional signaling pathways. The three-fold rise in serum sIL-6R levels during injury raises the possibility that IL-6 trans-signaling plays a part in AKI (Chen et al. 2019).

Chrusciel et al. (2014) found that trimetazidine has a protective impact on the kidneys by inhibiting inflammatory responses, reducing oxidative stress, avoiding apoptosis, and ameliorating endothelial dysfunction. TMZ blocks the kidney tubule’s epithelial cells (Yang et al. 2020).

By Atanasov et al. (2021), natural compounds and their structural counterparts have historically proven a highly helpful in pharmacotherapy, particularly for cancer and infectious disorders. From traditional medicinal plants and dietary supplements, the pharmaceutical professional of today can derive a wide range of necessary pharmaceuticals. Numerous medicinal plants are used throughout history due to their reputation as anti-inflammatory and anti-cytolytic agents (Chavan and Aniket 2019; Mohammed et al. 2021).

CBD’s supplementary significant marks in inflammation are the nuclear receptor Peroxisome Proliferator-Activated Receptor Gamma (PPARγ) (Granja et al. 2012). Co-administration of cannabidiol oil (natural product) caused downregulation to expression of IL6R that represented the anti-inflammatory property to CBD oil and its capability to attenuate renal toxicity in line with medication (dexamethasone and vitamin D), which partially accounts for their anti-inflammation to suppress IL-6 expression (Sanchez-Niño et al. 2012); any drug that can reduce the toxicity of DOX will be beneficial in such clinical disorders (Jagetia and Hmingthazuali 2018).

## Conclusions and prospects for the future

The antioxidant and anti-inflammatory properties of cannabidiol oil may be the reason for its potential renal protective effects in comparison with other drugs (trimetazidine). Biochemical, oxidative, and histological investigations of cannabidiol demonstrated its antioxidant activity and kidney protection.

### Supplementary Information

Below is the link to the electronic supplementary material.

Supplementary file 1 (spv 45.2 KB)

Supplementary file 2 (pdf 27.1 KB)

Supplementary file 3 (pdf 27.0 KB)

Supplementary file 4 (pdf 24.2 KB)

Supplementary file 5 (xlsx 10.2 KB)

Supplementary file 6 (pdf 26.5 KB)

Supplementary file 7 (pdf 23.0 KB)

Supplementary file 8 (pdf 26.5 KB)

Supplementary file 9 (docx 271 KB)

Supplementary file 10 (docx 15.7 KB)

## Data Availability

The raw data is available as supplementary data to this paper.
