# Supplementary material for: Prospective affirmative therapeutics of cannabidiol oil mitigates doxorubicin-induced abnormalities in kidney function, inflammation, and renal tissue changes
Source: Naunyn Schmiedebergs Arch Pharmacol. 2023 Nov 16;397(6):3897–906. doi: 10.1007/s00210-023-02836-4 (PMC11111484; doi:10.1007/s00210-023-02836-4)

Report on PCR results

21/12/2022

Settings of analysis: method: Threshold (Ct) (BFA), cr=9, vt=10, tp=30, tv=5

Date: 21 ПнУгИС 2022, 15:37:14
Protocol number: 0
Operator: Guest
Run file: Last_RUN.r48
Comment:

Test: **SYPERGREEN**

Amplification program: **SYPERGREEN (20µl)**

1. 94.0 °C - :02:00 X
2. 94.0 °C - :00:20 X
   60.0 °C - :00:30 X
   72.0 °C - :00:30 X

*40

Qualitative analysis

Number of the well	ID of the tube	Ct, Fam	Ct, Hex	Result
E2	1 (SYPERGREEN)	12.0		+
E3	2 (SYPERGREEN)	14.8		+
E4	3 (SYPERGREEN)	12.5		+
E5	4 (SYPERGREEN)	15.4		+

* Manual(Threshold) method (B,F,A) Threshold_FAM = 21.1 Threshold_HEX = 0.(

Dependence of FAM channel fluorescence on cycle number

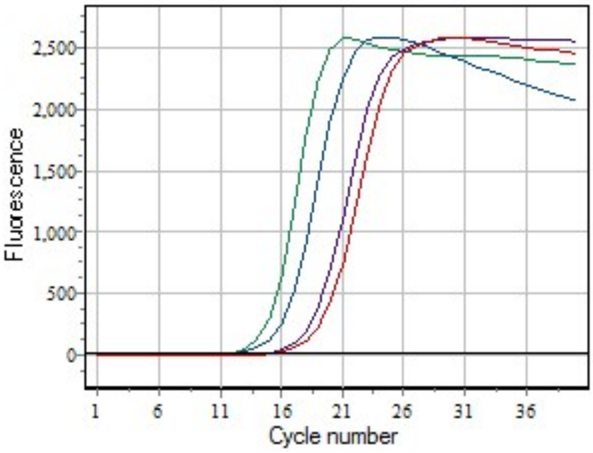

Supplement: Supplementary file 3 — (pdf 27.0 KB) [file 210_2023_2836_MOESM3_ESM.pdf]
