# Supplementary material for: Prospective affirmative therapeutics of cannabidiol oil mitigates doxorubicin-induced abnormalities in kidney function, inflammation, and renal tissue changes
Source: Naunyn Schmiedebergs Arch Pharmacol. 2023 Nov 16;397(6):3897–906. doi: 10.1007/s00210-023-02836-4 (PMC11111484; doi:10.1007/s00210-023-02836-4)

Report on PCR results

23/12/2022

Settings of analysis: method: Threshold (Ct) (BFA), cr=9, vt=10, tp=0, tv=0

Date: 23 ПнУгИС 2022, 17:03:01
Protocol number: 0
Operator: Guest
Run file: drnahla replicate.r48
Comment:

Test: **SYPERGREEN**

Amplification program: **SYPERGREEN3 (20µl)**

1. 94.0 °C - :02:00 X
2. 94.0 °C - :00:20 X
   60.0 °C - :00:30 X] *40
   72.0 °C - :00:30 X
3. 35.0 °C - :00:15 X
4. 35.0 °C - :00:15 X Δt] *55 (1.00)

Qualitative analysis

Number of the well	ID of the tube	Ct, Fam	Ct, Hex	Result
F3	1 (SYPERGREEN)	19.5		+
F4	2 (SYPERGREEN)	24.4		+
F5	3 (SYPERGREEN)	23.9		+
F6	4 (SYPERGREEN)			-

* Manual(Threshold) method (B,F,A) Threshold_FAM = 19.3 Threshold_HEX = 0.(

Dependence of FAM channel fluorescence on cycle number

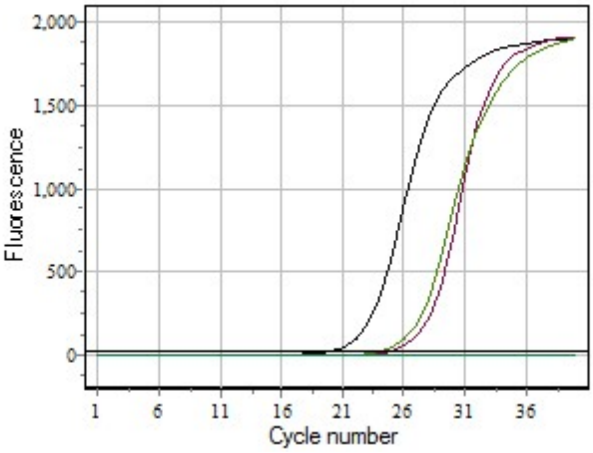

Supplement: Supplementary file 4 — (pdf 24.2 KB) [file 210_2023_2836_MOESM4_ESM.pdf]
