# Supplementary material for: Prospective affirmative therapeutics of cannabidiol oil mitigates doxorubicin-induced abnormalities in kidney function, inflammation, and renal tissue changes
Source: Naunyn Schmiedebergs Arch Pharmacol. 2023 Nov 16;397(6):3897–906. doi: 10.1007/s00210-023-02836-4 (PMC11111484; doi:10.1007/s00210-023-02836-4)

Report on PCR results

23/12/2022

Settings of analysis: method: Curve Shape (Cp) (BF), cr=9, vt=10, tp=30, tv=5

Date: 21 ПнУгИС 2022, 13:34:37
Protocol number: 0
Operator: Guest
Run file: DR NAHLA ABDEL FATAH.r48
Comment:

Test: **SYPERGREEN**

Amplification program: **SYPERGREEN 2 (20µl)**

1. 94.0 °C - :02:00 X
2. 94.0 °C - :00:20 X
   60.0 °C - :00:30 X
   72.0 °C - :00:30 X
   *40

Qualitative analysis

Number of the well	ID of the tube	Cp, Fam	Cp, Hex	Result
E2	1 (SYPERGREEN)	14.9		+
E3	2 (SYPERGREEN)	18.4		+
E4	3 (SYPERGREEN)	16.1		+
E5	4 (SYPERGREEN)	19.2		+

Dependence of FAM channel fluorescence on cycle number

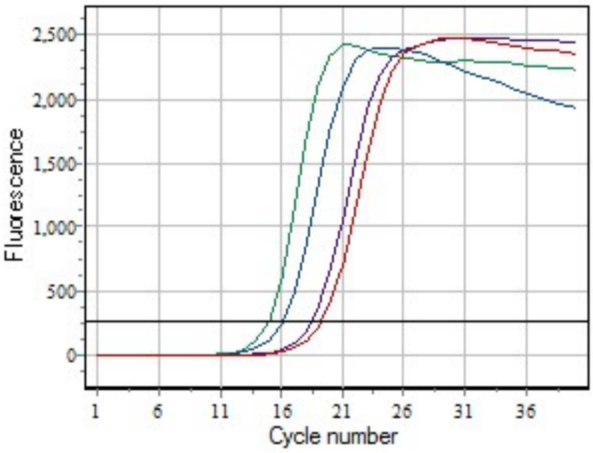

Supplement: Supplementary file 6 — (pdf 26.5 KB) [file 210_2023_2836_MOESM6_ESM.pdf]
