# Supplementary material for: Prospective affirmative therapeutics of cannabidiol oil mitigates doxorubicin-induced abnormalities in kidney function, inflammation, and renal tissue changes
Source: Naunyn Schmiedebergs Arch Pharmacol. 2023 Nov 16;397(6):3897–906. doi: 10.1007/s00210-023-02836-4 (PMC11111484; doi:10.1007/s00210-023-02836-4)

Report on PCR results

23/12/2022

Settings of analysis:	method: Threshold (Ct) (BFA), cr=9, vt=10, tp=0, tv=0
**Date:**	23 ПнУгИС 2022, 17:03:01
**Protocol number:**	0
**Operator:**	Guest
**Run file:**	drnahla replicate.r48
**Comment:**	

Test: **SYPERGREEN**

Amplification program: **SYPERGREEN3 (20µl)**

1. 94.0 °C - :02:00 X
2. 94.0 °C - :00:20 X
   60.0 °C - :00:30 X] *40
   72.0 °C - :00:30 X
3. 35.0 °C - :00:15 X
4. 35.0 °C - :00:15 X Δt] *55 (1.00)

Melting Curve

Number of the well	ID of the tube	Tm Fam	Tm Hex
F3	1 (SYPERGREEN)	81.6	

Dependence of FAM channel fluorescence on temperature

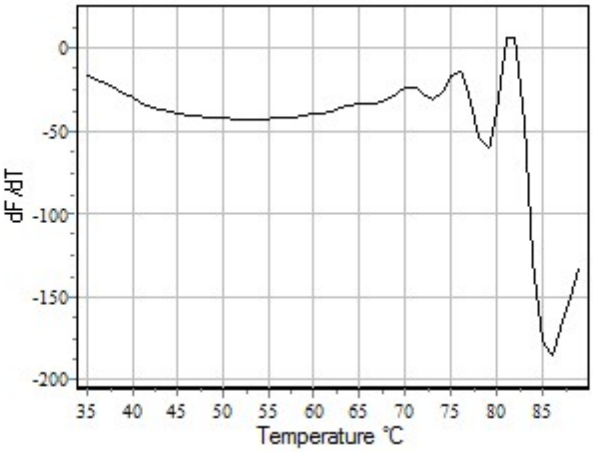

Supplement: Supplementary file 7 — (pdf 23.0 KB) [file 210_2023_2836_MOESM7_ESM.pdf]
