# Supplementary figures and images for: Prospective affirmative therapeutics of cannabidiol oil mitigates doxorubicin-induced abnormalities in kidney function, inflammation, and renal tissue changes
Source: Naunyn Schmiedebergs Arch Pharmacol. 2023 Nov 16;397(6):3897–906. doi: 10.1007/s00210-023-02836-4 (PMC11111484; doi:10.1007/s00210-023-02836-4)

**Graphical abstract**


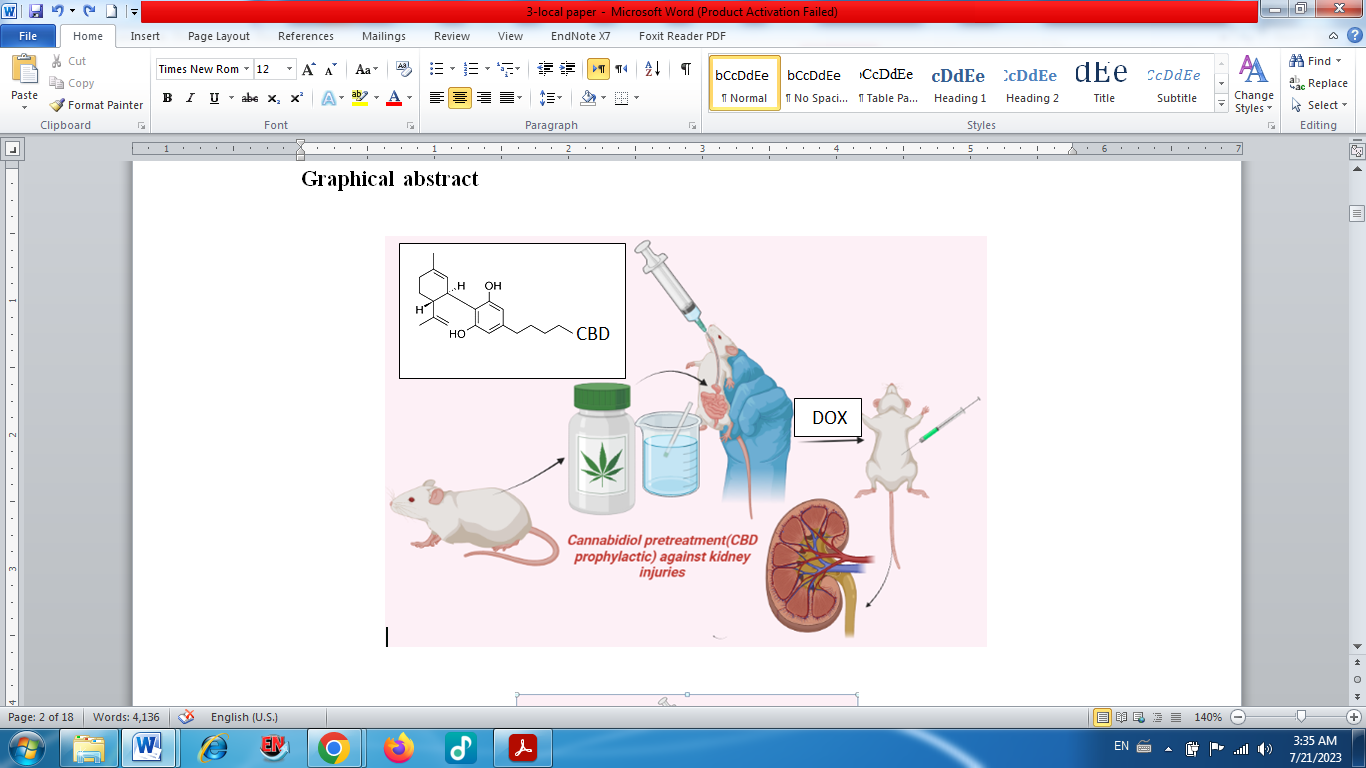

Supplement: Supplementary file 9 — (docx 271 KB) [file 210_2023_2836_MOESM9_ESM.docx]
