# Supplementary material for: Prospective affirmative therapeutics of cannabidiol oil mitigates doxorubicin-induced abnormalities in kidney function, inflammation, and renal tissue changes
Source: Naunyn Schmiedebergs Arch Pharmacol. 2023 Nov 16;397(6):3897–906. doi: 10.1007/s00210-023-02836-4 (PMC11111484; doi:10.1007/s00210-023-02836-4)
Supplement: Supplementary file 10 — (docx 15.7 KB) [file 210_2023_2836_MOESM10_ESM.docx]

**Prospective Affirmative Therapeutics of Cannabidiol Oil Mitigates Doxorubicin-Induced Abnormalities in Kidney Function, Inflammation, and Renal Tissue Changes.**

Nabil A. Soliman^1^, Samih I. El Dahmy^2^, Amr A. Shalaby ^1^ and Khadija A. Mohammed ^1*^

^1^Zoology Department, Faculty of Science, Zagazig University, Sharkia, Egypt

^2^Pharmacognosy Department, Faculty of Pharmacy, Zagazig University, Sharkia, Egypt,

**^*^Corresponding author:** **-Dr.Nabil Abbas Ahmed Soliman**

Professor of physiology, Department of Zoology, Faculty of Science, Zagazig University, Egypt

Email: nabilsoliman54@yahoo.co Tel. 00201096380800

2- Dr.Samih Ibrahim El Dahm

Professor of pharmacogonsy Dept. Faculty of pharmacy, Zagazig University, Egypt

[elsameeh@gmail.com](mailto:elsameeh@gmail.com)

3 Professor of physiology, Department of Zoology, Faculty of Science, Zagazig University, Egypt

[amrshalaby62@hotmail.com](mailto:amrshalaby62@hotmail.com)

4-Khadija Abd El Hakim Mohammed, phD Student, zoology Dept,Faculty of Science,Zagazig *university*

khadijahakim666@gmail.com
